# Rebound-Like Inflammatory Activity Presenting as a Tumefactive Demyelinating Lesion after Teriflunomide Discontinuation: A Case Report

**DOI:** 10.1159/crn/abyag014

**Published:** 2026-10-05

**Authors:** Alpkağan Kale, Nermin Tepe

**Affiliations:** Department of Neurology, Balıkesir University Faculty of Medicine Hospital, Balıkesir, Turkey

**Keywords:** Multiple sclerosis, Teriflunomide, Treatment discontinuation, Rebound-like activity, Tumefactive demyelination

## Abstract

**Introduction:**

Rebound after teriflunomide discontinuation is uncommon and difficult to distinguish from ordinary disease reactivation without a documented pretreatment baseline. Tumefactive demyelination is a rarely reported post-cessation phenotype.

**Case Presentation:**

This report presents a 39-year-old woman with relapsing-remitting multiple sclerosis had no relapses, Expanded Disability Status Scale (EDSS) worsening, or new MRI lesions during approximately 5 years of teriflunomide treatment. She stopped treatment without medical supervision or an accelerated elimination procedure for pregnancy planning. Four months later, persistent headache prompted MRI, which showed a 2.6 × 2.6-cm right frontal periventricular tumefactive demyelinating lesion with edema, peripheral diffusion restriction, and mild ring enhancement, plus a new left cerebellar plaque. EDSS remained 0. Absolute lymphocyte count rose from 600 cells/μL at the last on-treatment visit to 1,700 cells/μL at presentation. Serum MOG-IgG and AQP4-IgG and CSF cytology were negative; a type 2 oligoclonal-band pattern was present, and targeted infectious screening showed no evidence of active infection. Teriflunomide concentration was <0.02 mg/L twice, 14 days apart. Intravenous methylprednisolone (1 g/day for 5 days) resolved the headache. Cladribine was then started, and 1-month MRI showed marked regression. EDSS remained 0 and no new or enhancing lesion appeared through 12 months.

**Conclusion:**

The tumor-like lesion exceeded the patient’s pretreatment radiological phenotype, but the absence of objective deficit, EDSS worsening, and a high lesion burden makes definitive rebound uncertain. Rebound-like post-cessation inflammatory activity is the most defensible description. Supervised treatment transition and clinical and MRI monitoring are important when teriflunomide is stopped.

## Introduction

Teriflunomide is an oral disease-modifying therapy (DMT) for relapsing forms of multiple sclerosis (MS). It selectively and reversibly inhibits dihydroorotate dehydrogenase, limiting de novo pyrimidine synthesis and the proliferation of activated T and B lymphocytes without functioning as a lymphocyte-trafficking or cell-depleting therapy [1].

There is no universally accepted definition of post-DMT rebound. The term is generally reserved for inflammatory activity that exceeds the patient’s pretreatment disease activity after treatment withdrawal. A recent ECTRIMS workshop review summarized proposed indicators: a severe relapse with sustained disability, at least three new T2 or gadolinium-enhancing lesions, or at least one new tumor-like lesion. The review also emphasized that the evidence and definitions remain heterogeneous [2]. Rebound is best established after withdrawal of natalizumab and sphingosine-1-phosphate receptor modulators. Evidence after teriflunomide withdrawal is limited to a small number of case reports and short case series [3–6].

We report a woman with long-standing clinical and radiological stability who developed a new tumefactive demyelinating lesion (TDL) and a separate cerebellar plaque 4 months after unsupervised teriflunomide discontinuation. Because the event was clinically subtle and did not cause Expanded Disability Status Scale (EDSS) worsening, we use the term “rebound-like post-cessation inflammatory activity” rather than definitive rebound.

## Case Presentation

A 39-year-old woman was diagnosed with relapsing-remitting MS in 2014. She initially received subcutaneous interferon beta-1a (44 μg three times weekly). During the 24 months before teriflunomide initiation, she had no relapses, her EDSS score was 0, and brain MRI showed four T2 lesions without gadolinium enhancement. The lesion distribution included infratentorial/cerebellar involvement; spinal MRI showed no cord lesion. Interferon beta-1a was changed to teriflunomide in May 2020 because of injection-site reactions, not because of breakthrough disease.

During approximately 5 years of teriflunomide therapy, she remained relapse-free with an EDSS score of 0. Annual brain MRI examinations showed no new or enhancing lesions. At the last outpatient assessment, 3 months before treatment cessation, neurological examination and EDSS remained normal and the annual MRI was unchanged. Absolute lymphocyte counts at the last three on-treatment visits were 800, 650, and 600 cells/μL, respectively.

For pregnancy planning, the patient stopped teriflunomide without medical supervision, did not undergo an accelerated elimination procedure (AEP), and did not start a bridging or replacement DMT. Four months later, she presented with a mild persistent headache that was only partially responsive to analgesics. Neurological examination was normal and the EDSS score remained 0. Before corticosteroid treatment, the absolute lymphocyte count was 1,700 cells/μL.

Brain MRI, as documented in the final neuroradiology report, demonstrated a 2.6 × 2.6-cm right frontal periventricular lesion abutting the lateral ventricle, with prominent surrounding edema on fluid-attenuated inversion recovery (FLAIR), a peripheral rim of diffusion restriction, and mild peripheral ring enhancement (Fig. 1). A separate new left cerebellar plaque was also present. Spinal MRI showed no new lesion. Proton MR spectroscopy showed metabolite ratios within the laboratory reference ranges, and no neoplastic metabolic pattern was reported.

**Fig. 1.**
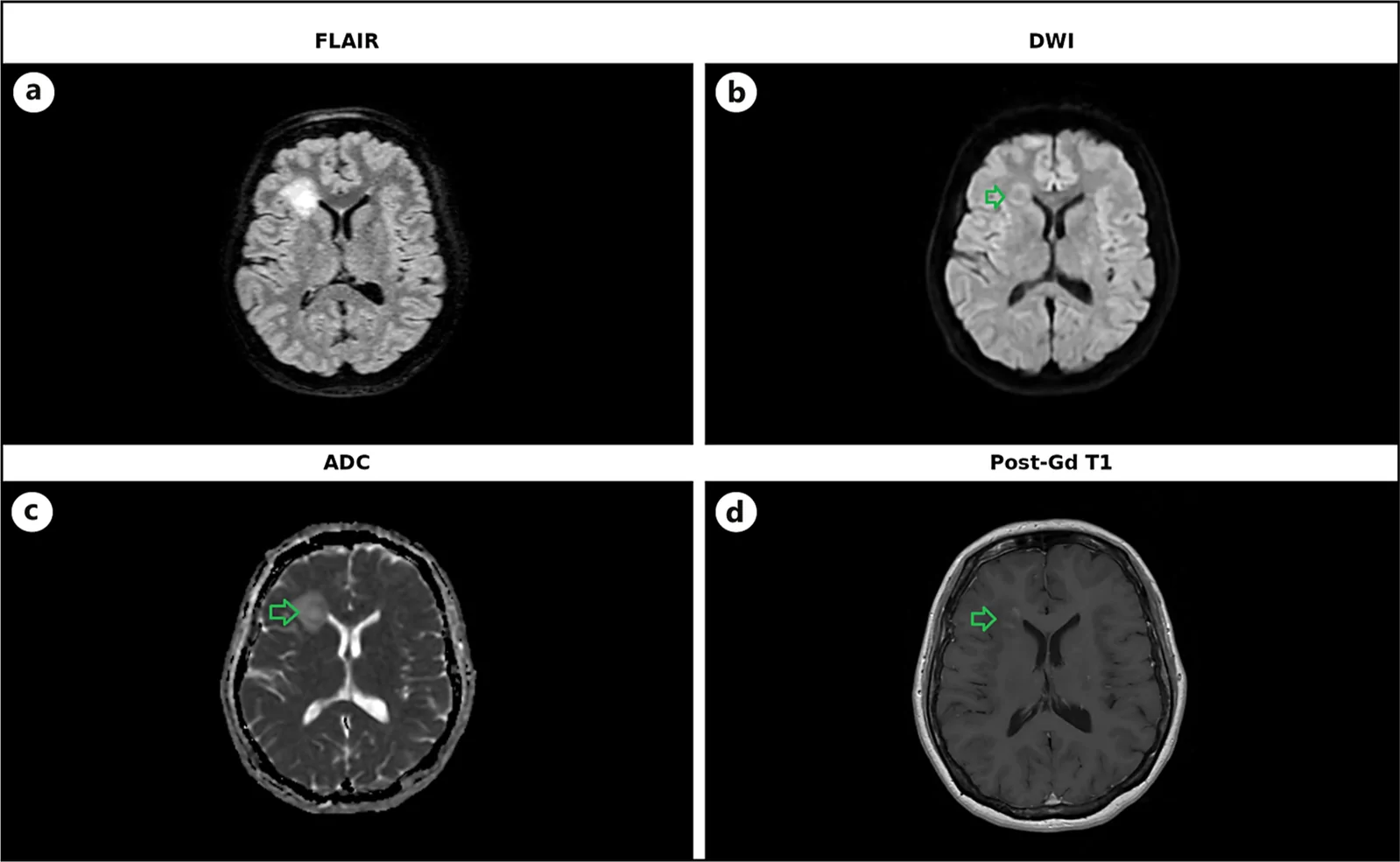
Axial brain MRI at presentation before corticosteroid treatment. **a** FLAIR shows a 2.6 × 2.6-cm right frontal periventricular lesion with surrounding edema. Diffusion-weighted imaging (DWI) (**b**) and apparent diffusion coefficient (ADC) map (**c**) demonstrate a peripheral rim of diffusion restriction. **d** Post-gadolinium T1-weighted imaging shows mild peripheral ring enhancement. Green arrows indicate the lesion in (**b–d**). These enhancement and diffusion findings reproduce the final neuroradiology report.

Alternative diagnoses were evaluated before corticosteroid exposure. C-reactive protein and erythrocyte sedimentation rate were within reference ranges. Cerebrospinal fluid studies showed no pleocytosis or protein elevation; cytology was negative for malignant cells, a type 2 oligoclonal-band pattern was present, and the IgG index was 0.9. Serum myelin oligodendrocyte glycoprotein immunoglobulin G (MOG-IgG) and aquaporin-4 immunoglobulin G (AQP4-IgG) were negative. Targeted infectious screening, including human immunodeficiency virus, syphilis, Lyme disease, tuberculosis, herpes simplex virus, viral hepatitis, and brucellosis, showed no evidence of active infection. There was no clinical or laboratory evidence of an active respiratory or urinary tract infection. Serum teriflunomide concentration was <0.02 mg/L and remained below this threshold on repeat measurement 14 days later. This threshold is used for embryofetal-risk management and is not a validated biomarker of protection from post-cessation MS activity [7, 8]. The patient received intravenous methylprednisolone 1 g/day for 5 days. The headache resolved completely.

Cladribine was started immediately after completion of corticosteroid treatment once standard pretreatment screening had excluded clinical contraindications. At 1 month after acute treatment, with cladribine initiated in the interval, MRI demonstrated marked lesion regression, resolution of the surrounding edema and diffusion restriction, and no residual contrast enhancement (Fig. 2), as documented in the final neuroradiology report. A brain biopsy was not performed because the known MS diagnosis, imaging pattern, negative alternative workup, and early radiological evolution converged on inflammatory demyelination. At 1-, 3-, 6-, and 12-month follow-up visits, neurological examination remained normal, EDSS was 0, and serial brain MRI showed no new or enhancing lesion. The clinical timeline is summarized in Table 1.

**Fig. 2.**
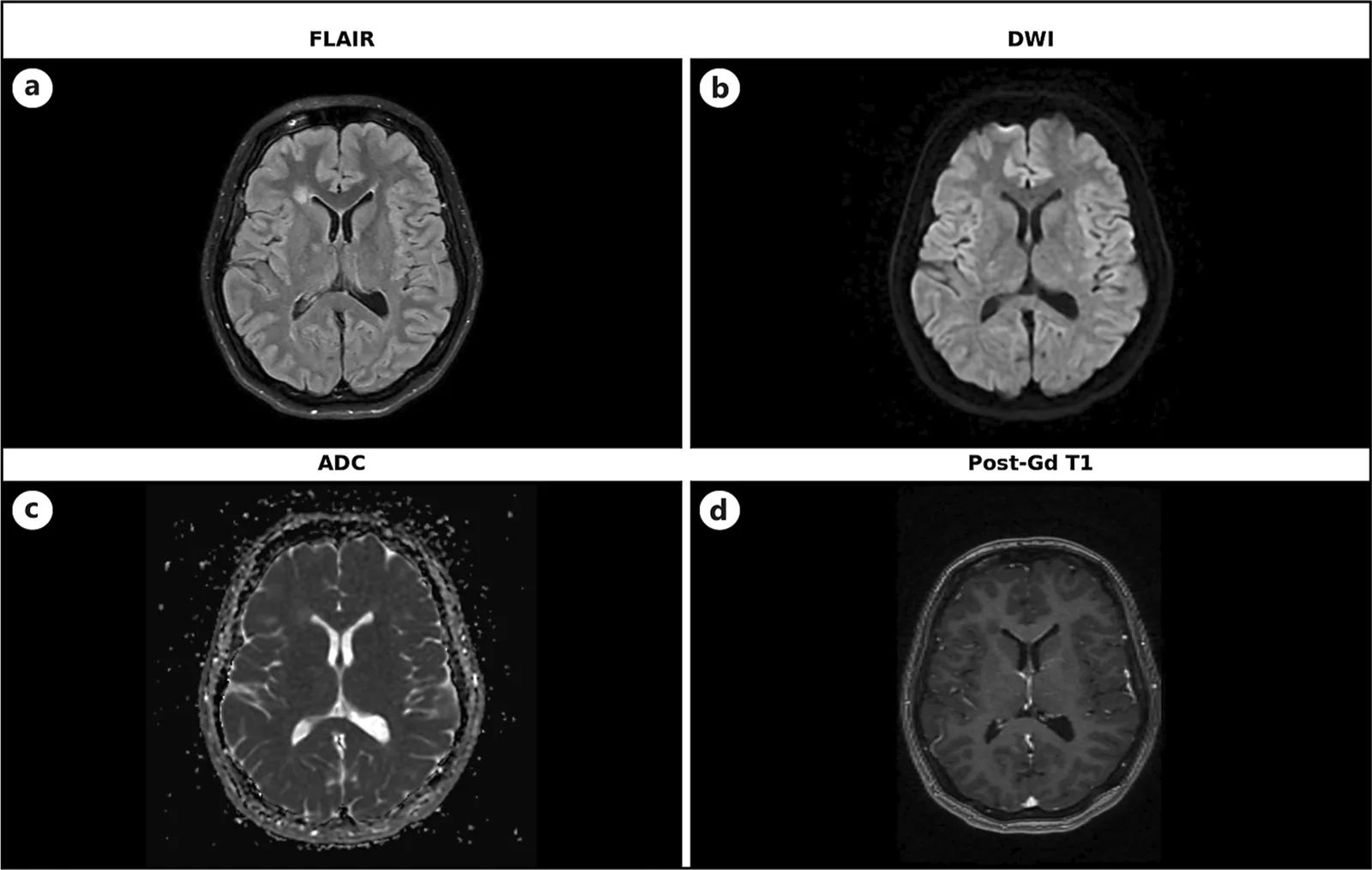
Approximately matched axial brain MRI at the lesion level 1 month after acute corticosteroid treatment; cladribine had been initiated in the interval. **a** FLAIR shows marked lesion regression with small residual hyperintensity and resolution of the surrounding edema. DWI (**b**) and ADC map (**c**) show no residual diffusion restriction. **d** Post-gadolinium T1-weighted imaging shows no residual enhancement, consistent with the final neuroradiology report.

**Table 1.** Clinical timeline

Time	Clinical and radiological event
2014	RRMS diagnosed; interferon beta-1a 44 μg three times weekly started
24 months before teriflunomide	No relapses; EDSS 0; four brain T2 lesions, no gadolinium enhancement; infratentorial/cerebellar involvement; no spinal cord lesion
May 2020	Switched to teriflunomide for injection-site reactions, not disease activity
Approximately 5 years on teriflunomide	No relapses; EDSS 0; annual MRI stable. Last three ALCs: 800, 650, and 600 cells/μL
3 months before cessation	Neurological examination normal, EDSS 0, annual MRI unchanged
Teriflunomide cessation	Unsupervised discontinuation for pregnancy planning; no AEP or replacement DMT
4 months after cessation	Persistent headache; examination normal; EDSS 0; ALC 1,700 cells/μL; right frontal TDL plus new left cerebellar plaque; teriflunomide <0.02 mg/L twice, 14 days apart
Acute treatment	Intravenous methylprednisolone 1 g/day for 5 days; headache resolved
Immediately after corticosteroids	Cladribine started after pretreatment screening
1 month after acute treatment	After interval cladribine initiation, marked TDL regression; no residual enhancement or diffusion restriction
1, 3, 6, and 12 months	Neurological examination normal; EDSS 0; no new or enhancing MRI lesion

## Discussion

The principal interpretive question is whether this event represents ordinary disease reactivation or rebound. The patient’s pretreatment and on-treatment course was low-activity: no relapse in the 24 months before teriflunomide, no relapse during 5 years of treatment, EDSS 0, and no new or enhancing MRI lesion on annual surveillance. The post-cessation event comprised two new brain lesions, one of which was a 2.6-cm tumor-like lesion. It therefore qualitatively exceeded her previous radiological phenotype and meets the previously proposed “at least one new tumor-like lesion” marker summarized in the recent review [2]. However, it did not cause an objective neurological deficit, EDSS increase, or a high numerical burden of new lesions. Headache also means that the event was not asymptomatic. These features preclude a categorical diagnosis of rebound and support the more precise term rebound-like post-cessation inflammatory activity.

The reported teriflunomide cases form a small and heterogeneous evidence base. Yamout et al. [3] described severe brain and spinal inflammatory activity 12 weeks after cessation. Fuerte-Hortigón et al. [4] reported two relapses after AEP and confirmation of teriflunomide concentrations below 0.02 mg/L, including a tumefactive presentation. Labauge et al. [5] subsequently reported two further cases after cessation. Polat et al. [6] described optic neuritis and multiple new enhancing lesions during pregnancy after AEP and low drug concentrations. The present case differs by the absence of AEP, spontaneous attainment of a low teriflunomide concentration, minimal symptoms, unchanged EDSS, and a dominant solitary TDL with one additional cerebellar plaque. We therefore do not claim that this is the first tumefactive case; its contribution is the clinically subtle phenotype documented against a well-characterized stable baseline.

A biologically plausible mechanism remains unproven. Teriflunomide suppresses proliferation of activated lymphocytes but does not sequester them in lymphoid tissue [1]. In this patient, the absolute lymphocyte count increased from 600 cells/μL at the last on-treatment assessment to 1,700 cells/μL at presentation. The temporal association is compatible with recovery of peripheral immune-cell availability after drug withdrawal, but total lymphocyte counts do not measure antigen specificity, functional activation, or CNS trafficking. The count change should therefore be viewed as supportive context rather than causal evidence.

A teriflunomide concentration <0.02 mg/L is a regulatory threshold intended to minimize embryofetal exposure, and repeat testing at least 14 days apart is recommended in European product information [7, 8]. It is not a neurologic safety threshold. Previous relapses after AEP and concentrations below this value further show that pharmacokinetic clearance does not eliminate the risk of inflammatory return [4, 6]. In the present case, AEP was not a trigger because it was not performed. The clinically relevant exposure was the unplanned treatment gap without an alternative DMT.

The lesion also required a careful tumefactive differential diagnosis. TDLs may mimic high-grade glioma, primary CNS lymphoma, abscess, and other inflammatory demyelinating disorders. Imaging features favoring TDL include limited mass effect relative to lesion size, incomplete or peripheral ring enhancement, peripheral rather than central diffusion restriction, and remote white-matter lesions [9, 10]. In this patient, the periventricular location, peripheral diffusion restriction, mild ring enhancement, concurrent new cerebellar plaque, established MS, negative MOG-IgG/AQP4-IgG, a type 2 oligoclonal-band pattern, infectious screening without evidence of active infection, and MR spectroscopy with metabolite ratios within reference ranges all favored demyelination. The oligoclonal-band pattern was compatible with the established MS diagnosis but was not specific for the tumefactive lesion. Proton MR spectroscopy can add discriminatory information but is not definitive [11].

Early regression at 1 month and sustained radiological stability for 12 months further supported the working diagnosis. The headache resolved after intravenous methylprednisolone, but cladribine was initiated before the 1-month MRI; the radiological improvement therefore cannot be attributed solely to corticosteroids. Corticosteroid responsiveness is also not pathognomonic: primary CNS lymphoma cells may partially or completely disappear after corticosteroid exposure and can mimic inflammatory demyelination [12]. Contemporary TDL series and a 2026 meta-analysis show that corticosteroids are commonly effective, but they do not substitute for longitudinal diagnostic vigilance [13, 14]. Accordingly, the absence of histopathological confirmation remains a limitation. Biopsy would be reconsidered if the lesion enlarged, enhancement recurred, or the clinical or imaging course became atypical.

Predictive factors for post-cessation activity are better defined for some DMT classes than for teriflunomide. Large observational data across DMTs associate younger age, female sex, and greater recent pretreatment activity with higher risk, whereas prompt initiation of a subsequent therapy reduces risk [15]. Our patient was female and relatively young but had no recent relapse, no enhancing lesion, and EDSS 0 before cessation. Thus, presently available clinical predictors would not have clearly identified her as high risk, and no teriflunomide-specific predictive model exists.

After acute treatment, cladribine was selected as the subsequent DMT and the patient remained clinically and radiologically stable through 12 months. Because this is a single case, neither the corticosteroid response nor the subsequent stability can establish causality between teriflunomide withdrawal and the TDL, quantify incidence, or identify the optimal post-cessation strategy. Additional limitations include the absence of biopsy, lymphocyte subset or functional studies, teriflunomide measurements between cessation and presentation, and the inability to separate the contribution of early cladribine initiation from corticosteroids to the 1-month MRI evolution.

## Conclusion

In a patient with 5 years of clinical and MRI stability, unsupervised teriflunomide discontinuation was followed 4 months later by a new TDL and a separate cerebellar plaque, with headache but no objective deficit or EDSS increase. The tumor-like lesion qualitatively exceeded the pretreatment phenotype, but the limited clinical severity and lesion number make definitive rebound uncertain. The term rebound-like post-cessation inflammatory activity best reflects the available evidence. Pregnancy planning should include supervised teriflunomide cessation, a defined transition strategy, and clinical and MRI monitoring; low plasma teriflunomide concentrations should not be interpreted as protection from renewed MS activity.

## Acknowledgments

We thank the patient for providing consent for publication. A large language model (ChatGPT, OpenAI) was used solely for language-editing assistance. The authors independently verified all clinical statements, images, citations, and interpretations and take full responsibility for the manuscript.

## Statement of Ethics

Ethical approval was not required for this single-patient case report in accordance with local guidelines and institutional policies. Written informed consent was obtained from the patient for publication of this case report and the accompanying images. The CARE checklist is submitted as online supplementary material (for all online suppl. material, see https://doi.org/10.1159/crn/abyag014).

## Conflict of Interest Statement

The authors have no conflicts of interest to declare.

## Funding Sources

This study was not supported by any sponsor or funder.

## Author Contributions

Alpkağan Kale: conceptualization, investigation, data curation, writing – original draft, and visualization. Nermin Tepe: supervision, validation, and writing – review and editing. Both authors approved the final manuscript.

## Data Availability

All data generated or analyzed during this case report are included in this article and its online supplementary material. Further inquiries can be directed to the corresponding author.
